# Hunting for the cause: Evidence for prion-like mechanisms in Huntington’s disease

**DOI:** 10.3389/fnins.2022.946822

**Published:** 2022-08-24

**Authors:** Kirby M. Donnelly, Cevannah M. Coleman, Madison L. Fuller, Victoria L. Reed, Dayna Smerina, David S. Tomlinson, Margaret M. Panning Pearce

**Affiliations:** ^1^Department of Biological Sciences, University of the Sciences, Philadelphia, PA, United States; ^2^Department of Biology, Saint Joseph’s University, Philadelphia, PA, United States

**Keywords:** Huntington’s disease, mutant huntingtin, polyglutamine, prion-like transmission, protein aggregate, aggregate spread, aggregate seed

## Abstract

The hypothesis that pathogenic protein aggregates associated with neurodegenerative diseases spread from cell-to-cell in the brain in a manner akin to infectious prions has gained substantial momentum due to an explosion of research in the past 10–15 years. Here, we review current evidence supporting the existence of prion-like mechanisms in Huntington’s disease (HD), an autosomal dominant neurodegenerative disease caused by expansion of a CAG repeat tract in exon 1 of the *huntingtin (HTT)* gene. We summarize information gained from human studies and *in vivo* and *in vitro* models of HD that strongly support prion-like features of the mutant HTT (mHTT) protein, including potential involvement of molecular features of mHTT seeds, synaptic structures and connectivity, endocytic and exocytic mechanisms, tunneling nanotubes, and nonneuronal cells in mHTT propagation in the brain. We discuss mechanisms by which mHTT aggregate spreading and neurotoxicity could be causally linked and the potential benefits of targeting prion-like mechanisms in the search for new disease-modifying therapies for HD and other fatal neurodegenerative diseases.

## Introduction

**FIGURE 1:**
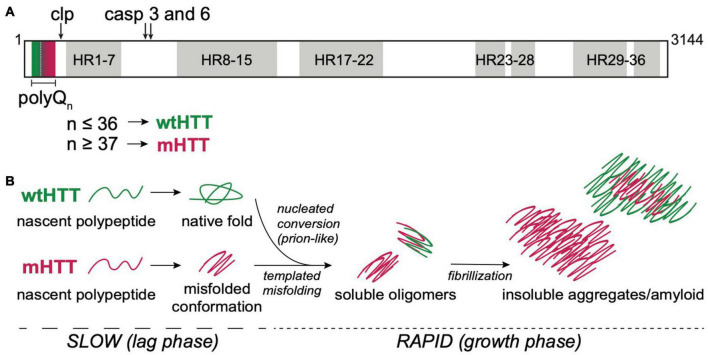
HTT structure and aggregation mechanism. **(A)** Primary protein structure of full-length human HTT highlighting the N-terminal polyQ tract encoded by exon 1, calpain (clp) and caspase (casp) 3 and 6 cleavage sites, and 7 regions containing 36 HEAT repeat (HR). PolyQ tract lengths associated with wtHTT (*n* ≤ 36) or mHTT (*n* ≥ 37) proteins are indicated by green and red, respectively, in and below the protein structure. **(B)** mHTT aggregation occurs *via* nucleated growth polymerization. wtHTT proteins achieve their native, functional fold, whereas expanded polyQ tracts cause mHTT proteins to misfold and a stabilize once a critical nucleus is achieved. This rate-limiting step is followed by rapid addition of mHTT monomers *via* templated misfolding to form soluble oligomers and, ultimately, insoluble, β-sheet-rich, amyloid fibrils. Prion-like conversion of wtHTT also occurs *via* templated conformational stabilization of natively-folded wtHTT proteins by mHTT aggregate seeds.

Huntington’s disease (HD) is a rare monogenic neurodegenerative disease characterized by motor, cognitive, and psychiatric deficits that typically develop in patients 30–50 years old and progress until death 10–15 years after clinical symptom onset. HD belongs to a family of nine dominantly-inherited neurodegenerative disorders collectively known as polyglutamine (polyQ) diseases, each caused by expansion of a CAG triplet repeat region that encodes a polyQ tract in a specific gene. HD is caused by expansion of a CAG repeat region in exon 1 of the *huntingtin* (*HTT*) gene located on chromosome 4 beyond a pathogenic threshold of at least 37 CAGs (Figure 1A), with inheritance of 40 or more CAGs in this stretch associated with 100% disease penetrance (Ross and Tabrizi, 2011; Bates et al., 2015; Saudou and Humbert, 2016). HD exhibits genetic anticipation due to increased instability of expanded CAG repeats, and there exists a strong inverse relationship between CAG repeat length and age of symptom onset, with inheritance of >60 CAG repeats associated with highly-aggressive, juvenile-onset HD (Duyao et al., 1993; Wexler, 2004). Treatments currently available to HD patients can temporarily relieve motor or psychiatric symptoms, but effective disease-modifying therapies have yet to be developed. Although HD is caused by inheritance of at least one mutant *HTT* allele, additional genetic factors that modify HD age-of-onset and severity are emerging (Gusella et al., 1983; Lee et al., 2015; Genetic Modifiers of Huntington’s Disease Consortium, 2019) and are being explored as potential therapeutic targets.

The mammalian HTT protein is large (>3,000 amino acid residues and ∼350 kDa; Figure 1A) and ubiquitously expressed, with highest levels detected in the central nervous system (CNS) and testes (Li et al., 1993; Guo et al., 2003). The CAG repeat expansion mutation that causes HD leads to expression of mutant HTT (mHTT) proteins containing expanded N-terminal polyQ tracts, which directly cause mHTT misfolding. Misfolded mHTT proteins accumulate in amyloid aggregates and appear as intranuclear inclusion bodies that are a defining diagnostic feature in HD brains. HTT misfolding and aggregation disrupt many cellular processes, and there is substantial evidence to suggest that mHTT oligomers and/or insoluble fibrils directly cause neurodegeneration in HD (Takahashi et al., 2008; Lajoie and Snapp, 2010; Leitman et al., 2013; Ramdzan et al., 2017). *HTT* knockout is embryonic lethal (Zeitlin et al., 1995), and conditional knockout leads to progressive neurodegenerative phenotypes in adult mice (Dragatsis et al., 2000), suggesting that *HTT* expression is required for normal development of the CNS. The majority of the large HTT protein consists of ten regions containing 36 HEAT repeats, named for HTT, Elongation factor 3, protein phosphatase 2A, and TOR1 (Figure 1A). Each HEAT repeat is ∼40 residues long and has a characteristic structure of two helical domains flanking a non-helical region. HEAT repeat-containing proteins adopt an overall solenoid-like structure that can accommodate dynamic interactions with many different proteins, suggesting primary functions as scaffolding proteins (Kobe et al., 1999). Compelling evidence points to a role for wild-type HTT (wtHTT) as a scaffold in selective autophagy (Ochaba et al., 2014; Rui et al., 2015), possibly accounting for accumulation of cytotoxic material due to loss of normal HTT function in HD. HTT has also been reported to play roles in transcriptional regulation, intracellular signal transduction, endocytosis, vesicle transport, and apoptotic signaling pathways by binding to over 350 different proteins (Saudou and Humbert, 2016). At least 45 amino acids, including >20 residues within the N-terminal domain encoded by exon 1, can be post-translationally modified by phosphorylation, acetylation, ubiquitination, sumoylation, fatty acylation, and/or proteolytic cleavage, suggesting complex and dynamic regulation of HTT functions in the cell (Saudou and Humbert, 2016). mHTT-induced neurodegeneration in HD likely involves loss-of-function phenotypes associated with HTT protein misfolding and sequestration in insoluble aggregates, and gain-of-toxic functions caused by dysregulation of protein-protein interactions or post-translational modifications, and/or disruption of key pathways that regulate cell homeostasis and survival (Ratovitski et al., 2012).

HTT proteins undergo post-translational processing by caspases, calpains, cathepsins, and matrix metalloproteinases that dramatically alter HTT function and/or subcellular localization (Figure 1A) (Weber et al., 2014). N-terminal fragments of mHTT generated by caspase cleavage (Graham et al., 2006) or aberrant splicing (Sathasivam et al., 2013; Neueder et al., 2017) are highly prone to oligomerizing, are cytotoxic, and have been identified in insoluble protein aggregates in *in vivo* HD models and HD patient brains, suggesting an active role for these fragments in disease pathogenesis (Myers et al., 1991; DiFiglia et al., 1997). Expression or exposure to aggregated N-terminal HTT fragments encoded by exon 1 (HTT_ex1_) or caspase-6 cleavage products (HTT_ex1–12_) are sufficient to induce neurotoxicity in cell, invertebrate, and vertebrate models (Wellington et al., 2000; Lunkes et al., 2002; Warby et al., 2008; Landles et al., 2010; El-Daher et al., 2015). Animal models expressing these N-terminal mHTT fragments recapitulate many of the behavioral and cellular pathologies seen in HD patients and have been invaluable for expanding our understanding of the molecular mechanisms that drive HD pathogenesis.

### Pathological aggregation of mutant huntingtin

HTT protein aggregation follows an amyloid nucleation mechanism, with oligomeric “seeds” that form during an extended lag phase, followed by rapid recruitment of HTT monomers into insoluble, β-sheet-rich fibrils during an exponential growth phase (Figure 1B) (Wetzel, 2020). The kinetics of mHTT amyloid formation are highly dependent on the length of the polyQ tract and flanking sequences contained within HTT_ex1_, especially the 17 residues N-terminal to the polyQ tract (Cooper et al., 1998; Thakur et al., 2009; Lakhani et al., 2010). The length of the aggregation lag phase can be substantially shortened by the addition of preformed HTT “seeds” or through secondary nucleation events, suggesting that mHTT aggregation is propagated through templated conversion of soluble monomers (Figure 1B). As for most neurodegenerative diseases, the identity of the toxic aggregate species in HD remains largely elusive. However, emerging evidence points to a strong correlation between soluble oligomers and increased pathogenicity (Takahashi et al., 2008; Leitman et al., 2013; Kim et al., 2016). Furthermore, formation of insoluble mHTT inclusions may actually serve a neuroprotective role by sequestering toxic mHTT proteins from key cell survival pathways (Arrasate et al., 2004; Slow et al., 2005).

Despite ubiquitous expression of *HTT*, there is striking regional development of neuropathology in the brains of HD patients. HD pathology is most prominent in the basal ganglia, where GABAergic medium spiny neurons (MSNs) in the striatum undergo massive degeneration, followed by degeneration of neurons in the cortex and other brain regions (Plotkin and Surmeier, 2015; McColgan et al., 2017). Intriguingly, striatal interneurons remain relatively spared as HD progresses, suggesting an intrinsic susceptibility of MSNs to mHTT-induced toxicity (Galvan et al., 2012; Morigaki and Goto, 2017). This selective vulnerability of MSNs likely results from a combination of mHTT-induced cell autonomous and non-cell autonomous toxicity, especially considering that mHTT expression is lower in the striatum than in brain regions that experience less degeneration in HD (Gourfinkel-An et al., 1997). In the striatum, mHTT expression and aggregation are associated with reduced brain-derived neurotrophic factor (BDNF) signaling and glutamate excitotoxicity, leading to loss of neurotrophic support and cortical input to MSNs (Deyts et al., 2009; McColgan and Tabrizi, 2018). Cell type-specific gene expression signatures and proteostasis regulation may also underlie the enhanced susceptibility of MSNs and other neuronal populations in HD. In support of this, MSNs express high levels of the GTPase and SUMO E3-like protein Rhes, which binds and sumoylates mHTT and is associated with increased cell death (Subramaniam et al., 2009). Thus, gene expression profiles and cell communication pathways that are differentially regulated in the brain may contribute to selective vulnerability of striatal and cortical neurons to HD pathogenesis.

### Prion-like transmission in neurodegenerative diseases

**FIGURE 2:**
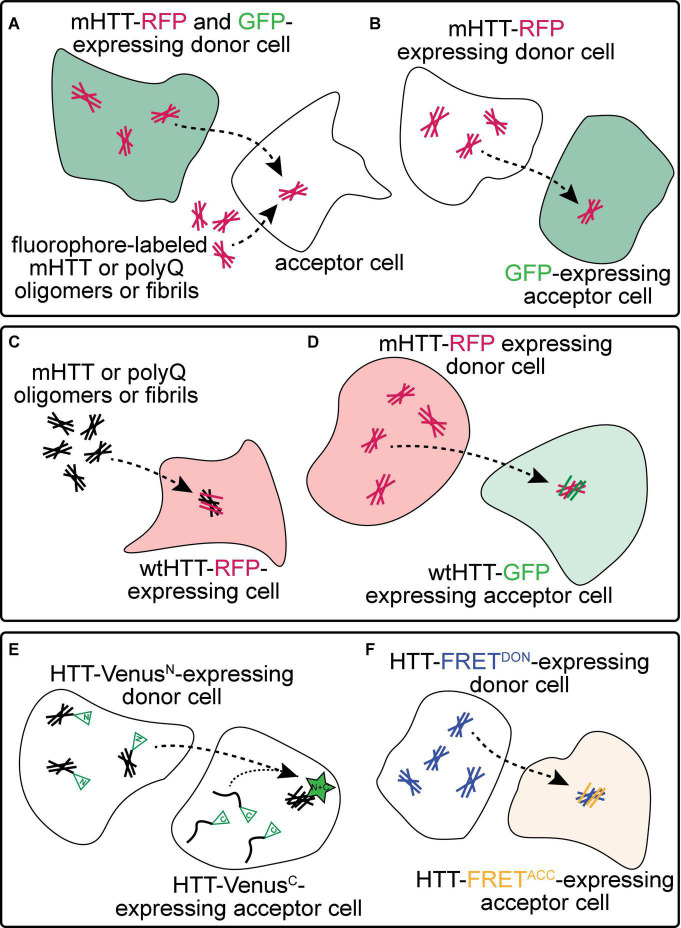
Experimental approaches to monitor prion-like behavior of mHTT proteins. **(A)** Spreading of mHTT can be reported as a time-dependent loss of co-localization between mHTT-FP expressed in “donor” neurons and a co-expressed non-transmissible cytoplasmic protein marker such as synaptophysin- or synaptotagmin-GFP. This approach has been used to monitor the transmissibility of mHTT proteins in mouse and fly brains (see Table 1). A similar approach *in vitro* monitors internalization of exogenous fluorescently-labeled mHTT or polyQ aggregates by several unlabeled “acceptor” cell types, including neuronal cell lines (e.g., SH-SY5Y, Neuro2A, and PC12), COS-7 fibroblast-like cells, and THP1 macrophages. **(B)** Transfer of mHTT-FP proteins from donor cells can also be detected by monitoring acquisition of mHTT-GFP signal within the cytoplasm of acceptor cells labeled by a soluble FP such as GFP, BFP, or mCherry. This approach has been used to demonstrate cell-to-cell mHTT spreading between neurons in mouse brain slices and cultured neuronal or primary neuron cells. **(C)** Entry of extracellular polyQ or mHTT fibrils into the cytoplasm of numerous cell types (e.g., HEK, HeLa, and PC12) causes templated aggregation of wtHTT-FP proteins, detected by a phenotypic change in wtHTT-FP expression pattern (e.g., diffuse → punctate) or decreased solubility measured by biochemical methods. **(D)** The seeding capacity of mHTT-FP aggregates can be measured by examining the aggregation of cytoplasmic wtHTT-FP proteins in acceptor cells templated by mHTT-FP aggregate seeds from donor cells. This approach has been applied to *in vitro* (e.g., in co-cultured HEK cells) and *in vivo* (e.g., adult *Drosophila* brains) experimental systems. **(E,F)** Physical interaction between mHTT seeds and monomeric HTT proteins originating in donor and acceptor cells, respectively, has been reported using biomolecular fluorescence complementation (BiFC; **E**), where each HTT protein is fused to a non-fluorescent GFP fragment, or by fluorescence resonance energy transfer (FRET; **F**), where HTT is fused to FP FRET pairs (e.g., CFP/YFP or GFP/mCherry).

Deposition of amyloid aggregates in the brain is a pathological hallmark of all age-related neurodegenerative diseases, including Alzheimer’s disease (AD), Parkinson’s disease (PD), frontotemporal dementia (FTD), amyotrophic lateral sclerosis (ALS), and polyQ disorders such as HD and spinocerebellar ataxias. Misfolded proteins that form the core of these insoluble deposits are unique to each disease and share no obvious homology with one another, but aggregation in each case occurs by propagation of a disease-associated, amyloidogenic protein conformation *via* a self-templating mechanism (Dobson et al., 2020), similar to the process illustrated for HTT in Figure 1B. Initially, it was thought that templated aggregation occurs between homotypic molecules within individual cells; however, abundant evidence now strongly supports a unifying mechanism whereby pathogenic protein assemblies transmit the aggregated state *between* cells in a manner similar to infectious prions (Brettschneider et al., 2015; Jucker and Walker, 2018) (Figures 1B, 2). Prions are protein-only infectious agents that form due to conformational change in the cellular prion protein (PrP*^C^*) from its native state to the more stable scrapie form (PrP*^Sc^*) and cause “prion diseases,” rare but fatal neurodegenerative disorders that include scrapie in sheep, chronic wasting disease in deer, bovine spongiform encephalopathy in cows, and Creutzfeldt-Jakob disease in humans (Prusiner, 2013). Most cases of prion disease occur sporadically, but PrP*^Sc^* molecules can also be acquired (e.g., through ingestion of prion-containing material) or stabilized by autosomal dominant mutations in the *PRNP* gene (Colby and Prusiner, 2011; Peggion et al., 2020). In all cases, prions spread by nucleated aggregation of PrP*^C^* monomers by PrP*^Sc^* “seeds” originating in other cells or even other organisms (Colby and Prusiner, 2011; Prusiner, 2013).

The idea that protein assemblies associated with neurodegenerative disorder have “prion-like” properties—i.e., nucleated aggregation of pathological proteins within and between cells—provides a convincing mechanism for the spatiotemporal patterns of aggregate pathology observed in AD, PD, and ALS patient brains (Braak and Braak, 1991; Braak et al., 2003; Brettschneider et al., 2013) and accelerated development of aggregate pathology in fetal neuron grafts transplanted into the brains of PD and HD patients (Kordower et al., 2008; Li et al., 2008; Cicchetti et al., 2014). Studies focused on expanding our understanding of molecular mechanisms underlying prion-like propagation of pathogenic protein aggregates formed by mHTT, tau, α-synuclein, TDP-43, and SOD1, and how aggregate spread is causally linked to neuronal loss, will lend insight into new therapeutic strategies that can impede progression of these fatal neurodegenerative diseases.

### Evidence for prion-like transmission in Huntington’s disease

Vast experimental evidence from the last ∼15 years supports the prion-like hypothesis for nearly all neurodegenerative diseases, but how aggregate spreading relates to neuropathogenesis in inherited disorders like HD, other polyQ diseases, and familial forms of AD, FTD, PD, and ALS is still unclear. As noted earlier, selective vulnerability of certain neuron populations to protein aggregate pathology is a defining feature of all neurodegenerative diseases, despite widespread expression of pathological proteins in the brain (Fu et al., 2018). Protein aggregate burden is strongly correlated with loss of neurons in affected brain regions, but there are exceptions to this rule, suggesting that multiple pathogenic mechanisms are at play. For example, in HD, the striatum is not the predominant site for either mHTT expression or inclusion formation, and thus mHTT protein enrichment alone cannot account for the selective degeneration of MSNs in the brain (Landwehrmeyer et al., 1995; Sapp et al., 1997). Enhanced susceptibility of MSNs to HD neuropathology must therefore involve other cell-intrinsic or non-cell autonomous factors. Intriguingly, network modeling of neuroanatomical changes in HD patient brains recapitulates the observed regional atrophy and supports a model of pathology spread along the structural connectome (Poudel et al., 2019; Raj and Powell, 2021). Prion-like transmission of neurotoxic mHTT seeds could therefore be an important contributor to HD progression by driving movement of neurotoxic mHTT seeds between anatomically-connected brain regions, possibly from cells that are less tolerant to mHTT-induced neurotoxicity to those that are more tolerant. Evidence obtained from post-mortem and *in vivo* human studies indicates that early disruption of cortical neuron structure and function precedes loss of MSNs, suggesting progression of toxic factors along vulnerable cortico-striatal connections (Rosas et al., 2005; Unschuld et al., 2012; Reiner and Deng, 2018). In BACHD mice, selective reduction of full-length mHTT protein expression in the cortex improves motor and psychiatric functions, whereas reducing mHTT expression in both the cortex and striatum rescues behavioral, tissue atrophy, and cortico-striatal synaptic defects (Wang et al., 2014). These findings raise the intriguing possibility that prion-like mHTT aggregate transmission along susceptible cortico-striatal synaptic paths in the brain may underlie HD neuropathogenesis.

Several lines of evidence support the idea that mHTT aggregates exhibit prion-like properties in HD patients. First, mHTT aggregates appeared in fetal striatal tissue grafts transplanted into three patients with manifest HD between 9 and 12 years prior to their death (Cicchetti et al., 2014). In post-mortem brain analyses, mHTT aggregates were found to associate with markers extracellular matrix in the grafted tissue as well as the host tissue, suggesting that mHTT secretion into the extracellular space could mediate its spread and/or toxicity. This finding was reproduced in wild-type mice that received xenografts derived from HD patient fibroblasts, where the transmitted mHTT aggregates led to HD-like neurodegenerative phenotypes in the mice (Jeon et al., 2016; Maxan et al., 2018). Second, pathological mHTT proteins are found in the cerebrospinal fluid (CSF) and blood of HD patients, with levels that correlate strongly with worsening motor and behavioral symptoms (Wild et al., 2015; Rieux et al., 2020). mHTT isolated from the CSF of HD subjects or BACHD rats is seeding-competent (Tan et al., 2015; Lee et al., 2020), suggesting that mHTT aggregates may propagate to peripheral tissues and mediate systemic HD pathologies (Chuang and Demontis, 2021). Heterogeneity in the structure and toxicity of mHTT aggregates (Shen et al., 2016; Ko et al., 2018; Mario Isas et al., 2021) implies the existence of mHTT strains that could be linked to variability in seeding ability or clinical HD phenotypes as seen for other amyloid aggregates (Tarutani et al., 2022). Interestingly, lesions formed by other pathogenic proteins, such as tau and α-synuclein, are also present in HD patient brains (Jellinger, 1998; Charles et al., 2000) and appear in fetal graft tissue (Cisbani et al., 2017; Ornelas et al., 2020), suggesting common pathways driving protein aggregation in these diseases. Anti-tau antibody treatment improves motor and cognitive performance in HD mice expressing mHTT_ex1_ (Alpaugh et al., 2022), suggesting active involvement of tau pathology in HD neuropathogenesis. Evidence for co-aggregation of mHTT proteins with other amyloidogenic proteins such as Aβ (Hartlage-Rübsamen et al., 2019) and TDP-43 (Coudert et al., 2019) suggests that heterotypic cross-seeding might also contribute to disease pathogenesis. Together, these findings point to the existence of shared molecular mechanisms and potential cross-talk between pathogenic proteins underlying these complex pathologies, raising the possibility that therapeutic approaches targeting protein aggregation could be effective against multiple proteopathies.

## Mechanisms underlying cell-to-cell transmission of mutant huntingtin

### Experimental models for prion-like spreading of mutant huntingtin

Numerous approaches have been used to monitor prion-like spreading of mHTT proteins in *in vitro* and *in vivo* models of HD and are summarized in Figure 2 and Table 1. Methods that report pathological protein transfer from “donor” cells to “acceptor” cells commonly use fluorescent labeling or fluorescent protein (FP)-fusions of HTT to track aggregate movement between cells *in vitro* or *in vivo*. The simplest of these approaches involves addition of fluorescently-labeled, exogenous aggregates formed from recombinant mHTT or polyQ peptides to unlabeled cells or tissues (Figure 2A) (Yang et al., 2002; Ren et al., 2009; Jeon et al., 2016; Ruiz-Arlandis et al., 2016; Masnata et al., 2019). Aggregate internalization is measured by detecting cell-associated mHTT signal using light microscopy or flow cytometry methods; however, these may lack sufficient resolution to distinguish intracellular vs. surface-bound HTT proteins. A modification of this approach improves this by detecting mHTT co-occurrence with a soluble, cytoplasmic protein marker such as GFP or mCherry expressed in the cytoplasm of donor (Figure 2A) (Pecho-Vrieseling et al., 2014; Babcock and Ganetzky, 2015) or acceptor cells (Figure 2B) (Costanzo et al., 2013; Pecho-Vrieseling et al., 2014; Sharma and Subramaniam, 2019). These approaches have been used in many experimental models to demonstrate mHTT aggregate entry into acceptor cells from the extracellular space or a donor cell cytoplasm. However, they fail to consider another key feature of prion-like proteins—the ability to recruit soluble versions of the same protein into aggregates. Expression of soluble wtHTT-FPs can address this and serves two purposes: (1) wtHTT marks the nucleocytoplasmic boundaries of acceptor cells, and (2) because wtHTT remains soluble at physiological concentrations unless nucleated by mHTT (Preisinger et al., 1999; Chen et al., 2001), induced aggregation of wtHTT-FPs reports the seeding capacity of prion-like mHTT species (Figures 2C–F) (Ren et al., 2009; Trevino et al., 2012; Pearce et al., 2015; Tan et al., 2015; Donnelly et al., 2020; Lee et al., 2020). Use of FP fusions for both mHTT and wtHTT proteins enhances spatial information by detecting co-localization between converted wtHTT proteins and their mHTT seeds (Figure 2D). Differentially-tagging mHTT and wtHTT proteins in donor and acceptor cells, respectively, has the added benefit of enabling bimolecular fluorescence complementation (BiFC; Figure 2E) (Lajoie and Snapp, 2010; Herrera et al., 2011; Kim et al., 2017) or fluorescence resonance energy transfer (FRET; Figure 2F) (Holmes et al., 2013; Pearce et al., 2015; Ast et al., 2018; Donnelly et al., 2020) approaches to measure direct molecular interaction of HTT proteins originating in different cells. Fusion of FP fragments commonly used in BiFC/split-FP approaches (Figure 2E) to HTT may also reduce interference of the tag with aggregation kinetics. Labeling multiple cell populations with different HTT proteins can be readily achieved in cultured cells by separately transfecting cell populations, but is more difficult in *in vivo* models. However, independent *in vivo* labeling of 2 or more cell types in the same tissue can be easily achieved in genetically tractable invertebrate models such as *Drosophila melanogaster* or *Caenorhabditis elegans*.

**TABLE 1:** Experimental approaches to study prion-like properties of HTT.

Experimental approach	Reference(s)	Summary of findings
Co-expression of mHTT with GFP in donor cells (Figure 2A)	Pecho-Vrieseling et al., 2014	mHTT_ex1_Q72 aggregates appeared in DARPP-32+ striatal MSNs following co-injection of viruses encoding mHTT_ex1_Q72 and synaptophysin-GFP into the cortex.
	Babcock and Ganetzky, 2015	mHTT_ex1–12_Q138-RFP aggregates spread from *Drosophila* ORNs labeled by synaptotagmin-GFP to large posterior neurons; patterns of spread depend on site of mHTT expression.
Addition of extracellular fluorescently-labeled mHTT aggregates to unlabeled acceptor cells (Figure 2A)	Yang et al., 2002	FITC-Q42 aggregates were internalized by PC-12 and COS7 cells by flow cytometry and confocal microscopy; addition of a nuclear localization sequence caused nuclear accumulation of the aggregates and increased cytotoxicity.
	Ren et al., 2009	Extracellular fibrils formed by FITC-K_2_Q44K_2_ peptides internalized by COS7 cells co-localize with intracellular markers of protein quality control systems (e.g., ubiquitin, proteasome subunits, and Hsp70).
	Jeon et al., 2016	Internalization of GFP-mHTT_ex1_Q103 from conditioned media containing exosomes derived from HEK cells into SH-SY5Y cells
	Ruiz-Arlandis et al., 2016	Alexa Fluor 488-mHTT_ex1_Q44 and FITC-K_2_Q44K_2_ fibrils were internalized by Neuro2A cells; internalization and seeding efficiency was higher in undifferentiated vs. differentiated Neuro2A cells.
	Masnata et al., 2019	ATTO488/550-mHTT_ex1_Q48 fibrils induced the aggregation of endogenous wtHTT in SH-SY5Y neuroblastoma cells and differentiated THP1 macrophages.
Expression of mHTT-FPs in donor cells and soluble cytoplasmic FPs in acceptor cells (Figure 2B)	Costanzo et al., 2013	GFP-mHTT_1–480_Q68 aggregates spread from donor mouse catecholaminergic neuronal (CAD) cells to acceptor CAD cells expressing soluble mCherry *via* TNTs; similar results observed in primary cerebellar granule neuron co-cultures.
	Pecho-Vrieseling et al., 2014	mHTT_ex1_Q150 aggregates transferred from R6/2 mouse organotypic brain slices to synaptically-connected human neurons derived from embryonic or induced pluripotent stem cells; mHTT_ex1_Q150 aggregates transferred from R6/2 cortex to wild-type striatum in mixed genotype brain slice co-cultures.
	Sharma and Subramaniam, 2019	Full-length mHTTQ111 and mCherry-mHTT_*N*171_Q89 aggregates transferred from GFP-Rhes-expressing striatal cells to co-cultured striatal neuron acceptor cells labeled with BFP through TNTs positive for Rhes-GFP; HTT transfer depended on its SUMOylation state.
Addition of unlabeled polyQ or mHTT fibrils or seeds to nucleate the aggregation of wtHTT-FP expressed in cells (Figure 2C)	Ren et al., 2009	Fibrils formed by K_2_Q44K_2_ peptides or recombinant mHTT_ex1_Q51 proteins added to the extracellular media induced aggregation of cytoplasmic wtHTT_ex1_Q25-mCherry in HEK cells.
	Trevino et al., 2012	Extracellular K_2_Q44K_2_, D_2_Q44D_2_, or acetylated- K_2_Q44K_2_ fibrils induced prion-like conversion of wtHTT_ex1_Q25-mCherry in HEK cells; Extracellular fibrils formed by K_2_Q44K_2_ peptides or recombinant mHTT_ex1_Q44 proteins seed the aggregation of wtHTT_ex1_Q25-mCherry in HeLa cells.
	Tan et al., 2015	Addition of extracellular K_2_Q44K_2_ fibrils or CSF samples from BACHD mice or postmortem or living HD patients enhances aggregation of mHTT_ex1_Q103-GFP in PC12 cells and cell-free lysates.
	Lee et al., 2020	mHTT_ex1_Q46-GFP aggregation in HEK cells was accelerated by addition of mHTT_ex1_Q51 fibrils and sonicated seeds. Mouse and human HD brain lysates and CSF samples induced aggregation of mHTT_ex1_Q46-GFP with the first 17 residues deleted (ΔN17).
Seeded aggregation of cytoplasmic HTT-FPs in acceptor cells by mHTT-FPs expressed in donor cells (Figure 2D)	Ren et al., 2009	Co-culturing mHTT_ex1_Q71-GFP- and wtHTT_ex1_Q25-mCherry-expressing HEK cells increased seeded aggregation of wtHTT_ex1_Q25-mCherry; enhanced by puromycin-mediated lysis of mHTT_ex1_Q71-GFP-expressing cells
	Pearce et al., 2015; Donnelly et al., 2020	mHTT_ex1_Q91-mCherry aggregates in *Drosophila* presynaptic ORNs induce prion-like conversion of wtHTT_ex1_Q25-GFP expressed in glia and in postsynaptic projection neurons (PNs); mHTT aggregates transferred *trans*-synaptically through glial intermediates.
	Ruiz-Arlandis et al., 2016	Seeded aggregation of cytoplasmic wtHTTex1Q25-mCherry by extracellular fluorophore-labeled HTTex1Q44 fibrils occurs more efficiently in undifferentiated than differentiated Neuro2A cells. Fibrils are internalized by clathrin-dependent endocytosis and co-localize with endosomal and lysosomal markers.
Expression of split-GFP/Venus or FRET-pair mHTT constructs in BiFC- and FRET-based approaches (Figures 2E,F)	Lajoie and Snapp, 2010	BiFC occurred between mHTT_ex1_Q145-GFP^N(or^ ^C)^ and wtHTT_ex1_Q23-GFP^C(or^ ^N)^ proteins after co-transfection of Neuro2A cells.
	Herrera et al., 2011	BiFC occurred between mHTT_ex1_Q103-Venus^N^ with mHTT_ex1_Q103-Venus^C^ proteins in co-cultures of H4 or HEK cells separately transfected with each construct.
	Holmes et al., 2013	Aggregation of co-expressed wtHTT_ex1_Q25 proteins fused to CFP or YFP induces FRET in C17.2 neural precursor cells treated with FITC-mHtt_ex1_Q50 fibrils; Unlike tau and α-synuclein, mHTT fibril internalization was independent of heparin sulfate proteoglycans.
	Kim et al., 2017	BiFC detected between wtHTT_ex1_Q25 and mHTT_ex1_Q97 constructs fused to Venus FP fragments and expressed in pharyngeal muscle cells and connected neurons.
	Pearce et al., 2015; Donnelly et al., 2020	FRET detected between FP tags on mHTT_ex1_Q91-mCherry and wtHTT_ex1_Q25-GFP expressed in donor and acceptor cells in a *Drosophila* HD model.
	Ast et al., 2018	Measurement of seeding-competent HTT species in biological samples using a FRET-based mHTT aggregate seeding (FRASE) assay; seeding activity was detected for small mHTT structures in presymptomatic HD mice; seeds are toxic in *Drosophila* HD model.
Host-to-graft spread of mHTT in HD patient brains	Cicchetti et al., 2014; Maxan et al., 2018	mHTT aggregates co-localized with the extracellular matrix marker phosphocan in grafted solid or suspension fetal tissues that had survived ∼10–15 years following transplantation in HD patient brains.
Mammalian focal injection models	Jeon et al., 2016	Appearance of mHTT aggregates in mouse striatum and cortex after injection of HD patient-derived fibroblasts (Q72, 143, or 180) or iPSCs (Q143) into the lateral ventricles. Injection of exosomes derived from HD fibroblasts led to neurological deficits and appearance of mHTT pathology in DARPP-32+ MSNs.
	Masnata et al., 2019	Intracerebral injection of mHTT_ex1_Q48 fibrils into wild-type or R6/2 mice produced behavioral and biochemical changes, most notably co-localization of mHTT proteins in R6/2 mouse brains with the exogenous mHTT_ex1_Q48 fibrils.
	Gosset et al., 2020	mHTT from HD-derived brain homogenates injected into wild-type or BACHD mouse cortex spread to sites distant from the injection site and worsened behavioral phenotypes only in BACHD mice; Injection of brain homogenates from a juvenile HD patient into the striatum of non-human primates caused persistence of mHTT near the injection site, but was not associated with neurological impairments.

**FIGURE 3:**
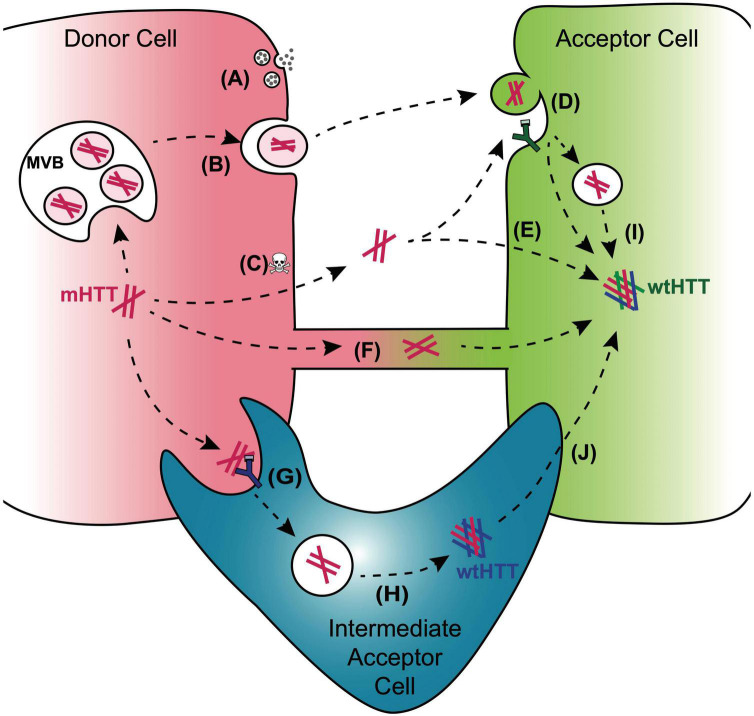
Mechanisms for cell-to-cell transmission of mHTT. Pathways reported to mediate inter-cellular transmission of mHTT aggregates are illustrated here and described in more detail in the text. mHTT aggregate release from donor cells (red cell; left) may be coupled to synaptic activity in presynaptic neurons **(A)**, could occur with in exosomes released from a multivesicular body (MVB) **(B)**, or could be passively released from dying cells **(C)**. Entry of prion-like mHTT aggregates into acceptor cells has been reported to occur *via* bulk-phase or receptor-mediated endocytosis **(D)**, direct penetration of the plasma membrane **(E)**, or alternatively, aggregates may transfer directly from one cell cytoplasm to another *via* membrane-enclosed tunneling nanotubes **(F)**. Phagocytic glia may play double-edged roles in HD through receptor-mediated engulfment of aggregates from neurons **(G)**, which can lead to either clearance in the lysosome or aggregate “escape” from the glial phagolysosomal system prior to degradation **(H)**. Disruption of normal endosomal or autophagosomal pathways may also underlie mHTT aggregate transmission to the cytoplasm of non-phagocytic cells **(I)**. mHTT aggregates that evade lysosomal degradation as a result of endo/phagolysosomal defects could generate cytoplasmic reservoirs of prion-like mHTT species in “intermediate acceptor cells” (e.g., glia) and enhance aggregate seed transmission to other cells, such as post-synaptic neurons **(J)**.

A combination of these strategies has been employed to investigate prion-like spreading of mHTT in multiple model systems, including cultured mammalian cells, *ex vivo* mouse brain slices, and *in vivo* approaches in *Caenorhabditis elegans*, *Drosophila*, mice, and non-human primates (Table 1). *In vitro* studies have revealed important information about the kinetics of mHTT aggregate transfer and subcellular localization of mHTT as it transits between individual cells. On the other hand, transgenic animal models of HD may accurately model mHTT transmission in tissues, where multiple cell types interact and communicate in complex ways that are not easily replicated in cultured cells. Together, these models have lent abundant insight into the molecular mechanisms that underlie prion-like mHTT spreading, including the molecular properties of seeding-competent mHTT aggregate species and the genes and pathways that mediate cell-to-cell aggregate transfer. The remainder of this review will summarize the mechanisms identified for inter-cellular mHTT aggregate transmission (summarized in Figure 3) and ways in which these findings can be utilized to improve therapeutic options for HD patients.

### *Trans*-synaptic transmission of mutant huntingtin

Synaptic dysfunction and loss are early features of all neurodegenerative diseases and typically precede detectable tissue atrophy or clinical manifestations (Schirinzi et al., 2016; Jackson et al., 2019; Smith-Dijak et al., 2019). Pathogenic protein aggregates directly interfere with many key synaptic functions, and prion-like mechanisms could be a primary driver of synaptotoxicity, particularly for vulnerable neurons and their pre- and postsynaptic partners. There is accumulating evidence to support *trans*-synaptic spread of many protein aggregates, including mHTT, Aβ, tau, and α-synuclein (Freundt et al., 2012; Dujardin et al., 2014; Brahic et al., 2016; Donnelly et al., 2020), and a role for neural activity in aggregate spreading (Pecho-Vrieseling et al., 2014; Babcock and Ganetzky, 2015; Wu et al., 2016, 2020; Donnelly et al., 2020). These findings suggest that synaptopathology, regional vulnerability, and aggregate transmissibility are intimately linked. Several research groups have explored the hypothesis that mHTT propagation is linked to neural connectivity in experimental HD models, with varying results that could reflect time-dependent or cell type- or regional- specificities. mHTT_ex1_ proteins transfer from R6/2 HD cortico-striatal slices to ectopically-connected human neurons, and this was inhibited by botulinum toxin treatment (Pecho-Vrieseling et al., 2014), suggesting a role for SNARE-dependent synaptic functions in mHTT spreading. In the same study, spreading of mHTT was also observed from R6/2 cortical to wild-type striatal neurons in mixed genotype cultures and following injection of wild-type mouse brain cortex with viruses encoding FP-tagged mHTT_ex1_, with increased appearance of mHTT_ex1_ protein in striatal neurons over time (Pecho-Vrieseling et al., 2014). More distant mHTT spreading was seen after focal injection of mouse striatum with purified polyQ fibrils or HD brain homogenates and was associated with worsening symptoms in both wild-type and BACHD mice (Masnata et al., 2019; Gosset et al., 2020). However, inoculation of the striatum of non-human primates with brain homogenates from a juvenile HD patient led to persistence of mHTT pathology near the injection site, but there was no evidence for mHTT spreading or behavioral abnormalities associated with the injected material (Gosset et al., 2020). Synaptosomal mHTT assemblies isolated from HD mouse brains possess higher seeding capacity than mHTT in ER/Golgi fractions (Chongtham et al., 2021), suggesting that the synaptic environment is favorable for mHTT propagation. Taken together, these studies indicate that genetic background, molecular features of mHTT aggregates, and/or context-specific sensitivities to mHTT toxicity influence progression of HD pathology in the brain.

Invertebrate HD models also support a role for synaptic activity in the spread of mHTT aggregates (Figure 3A). Two *Drosophila* models of HD support spreading of FP-tagged mHTT_ex1_ and mHTT_ex1–12_ proteins from olfactory receptor neurons (ORNs) to their postsynaptic partners, projection neurons (PNs) (Donnelly et al., 2020), or to neurons in more distant regions of the adult fly brain (Babcock and Ganetzky, 2015). Interestingly, inhibition of synaptic activity in mHTT-expressing ORNs had different effects on mHTT protein spreading in each model. In one study, stalling synaptic vesicle recycling in ORNs expressing RFP-mHTT_ex1–12_ proteins by expressing temperature-sensitive dynamin mutants decreased the appearance of transgenic mHTT proteins in neurons in other regions of the fly brain (Babcock and Ganetzky, 2015). In another study, inhibiting synaptic activity of mHTT_ex1_-expressing ORNs using either temperature-sensitive dynamin mutants or tetanus toxin subunits to block SNARE-mediated vesicle fusion enhanced the spread of mHTT_ex1_ aggregates to postsynaptic PNs (Donnelly et al., 2020). The molecular basis for these differing results remains to be determined, but could be due to unique molecular properties of pathogenic HTT_ex1_ vs. HTT_ex1–12_ protein fragments or roles for neural activity in tissue environments that support *trans*-synaptic vs. non-synaptic aggregate transmission. Together, these studies indicate that relationships between neural activity and mHTT aggregate spreading are complex and likely to be protein-, cell-, and context-specific.

### Cellular uptake and release of mutant huntingtin

Cells interact in dynamic ways with their extracellular environments, including actively internalizing and/or releasing material *via* diverse endocytic and exocytic processes. During endocytosis and the analogous process of phagocytosis, extracellular material is internalized *via* non-specific, “bulk-phase” processes or by directly activating cell surface receptors that initiate engulfment (Hu et al., 2015; Levin et al., 2016; Borchers et al., 2021). Exocytic processes mediate the release of cellular material to initiate cell-cell communication or eliminate toxic material from damaged cells (Quek and Hill, 2017). Proper functioning of endocytosis, phagocytosis, and exocytosis pathways is critical for brain homeostasis during development and adulthood, and thus it is not surprising that defects in many endocytic and exocytic pathways are implicated in the pathogenesis of many neurological disorders, including neurodegenerative diseases (Giovedì et al., 2020).

Entry of oligomeric and amyloid assemblies of mHTT into a cell’s cytoplasmic compartment from the extracellular space or from a different cell cytoplasm has been reported to occur *via* multiple endocytic pathways (Figure 3D). polyQ expansion and protein aggregation can alter the interaction of HTT with endocytic machinery components and lead to endocytic dysfunction (Parker et al., 2007; Davranche et al., 2011; Borgonovo et al., 2013). Genetic or pharmacological inhibition of receptor-mediated endocytosis, macropinocytosis, or the GTPase activity of dynamin reduces the ability of purified polyQ or mHTT_ex1_ fibrils to be internalized by multiple cultured mammalian cell types (Holmes et al., 2013; Ruiz-Arlandis et al., 2016), suggesting that mHTT can enter cells from the extracellular space *via* multiple endocytic routes. Treatment of cells with exogenous fibrils formed by mHTT_ex1_, SOD1, α-synuclein, or TDP-43 proteins triggers membrane ruffling, a phenomenon associated with Rac1-dependent macropinocytosis (Zeineddine et al., 2015) and suggests that extracellular aggregates activate endocytic pathways to stimulate internalization. Interestingly, aggregates formed by pathogenic polyQ_44_ peptides were observed by deep-etch transmission electron microscopy to be directly associated with the actin cytoskeleton in HEK cells, suggesting that fibrils have the ability to directly penetrate the plasma membrane bilayer (Ren et al., 2009) (Figure 3E). This property may also extend to intracellular membranes, as extracellular fibrils formed by mHTT_ex1_, tau, and α-synuclein damage intracellular vesicle membranes, indicated by co-localization with Galectin-3, in cultured neuroblastoma cells (Flavin et al., 2017). These findings suggest that a combination of active and passive mechanisms permit mHTT protein assemblies to gain entry to a cell’s cytoplasm where they can effect prion-like conversion of soluble HTT monomers.

Aggregation of mHTT and other pathological, disease-associated proteins is associated with proteostasis impairment in cells. Defects in the ability of cells to clear and post-translationally regulate mHTT proteins declines with age, accelerating aggregate formation and associated toxicity (Tsvetkov et al., 2013). HTT’s interactions with components of the autophagy pathway (Petersén et al., 2001; Atwal and Truant, 2008; Greco et al., 2022) and its role in selective autophagy (Ochaba et al., 2014; Rui et al., 2015) suggest that HTT loss-of-function could decrease clearance of aggregates and other toxic debris. Disruption of endolysosomal and autophagolysosomal functioning by mHTT could generate partially-degraded mHTT aggregates (Trajkovic et al., 2017), possibly directly contributing to dissemination of prion-like mHTT aggregates in the brain (Figures 3H,I). Interestingly, several studies have reported an inverse relationship between the size of mHTT seeds and their seeding capacity (Ast et al., 2018; Donnelly et al., 2020; Lee et al., 2020; Schindler et al., 2021). It is thus possible that fragmentation or incomplete degradation of larger mHTT aggregates could lead to the formation of small, highly transmissible seeds.

Release of mHTT from “donor” cells could occur *via* active mechanisms such as secretion or packaging in extracellular vesicles (EVs; Figure 3B), or passively during cell death (Figure 3C) (Brahic et al., 2016; Trajkovic et al., 2017; Wang et al., 2017; Caron et al., 2021). mHTT secretion is postulated to be neuroprotective by reducing the load of mHTT aggregates in cells (Lee et al., 2016; Deng et al., 2017; Hong et al., 2017), or neurotoxic, as a mode of aggregate spreading (Jeon et al., 2016; Zhang et al., 2016). Exosomes have recently emerged as a focus of attention in many fields of research as “shuttles” for transferring proteins, lipids, and RNA between cells. Exosomes are small EVs (∼50–100 nm in diameter) that form following invagination of a larger endosomal structure known as the multi-vesicular body, which fuses with the plasma membrane to shed exosomes (Kalani and Tyagi, 2015; van Niel et al., 2018). Exosomes package and transport signaling molecules such as proteins, mRNAs, and miRNAs between cells and across the blood–brain barrier as a key mode of cell-cell communication between diverse cells and tissues. Pathogenic proteins associated with most neurodegenerative diseases have been detected in exosomes secreted into the extracellular space by cultured cells or *in vivo*, including mHTT (Jeon et al., 2016; Zhang et al., 2016; Deng et al., 2017), Aβ (Rajendran et al., 2006), tau (Simón et al., 2012; Asai et al., 2015), α-synuclein (Emmanouilidou et al., 2010), SOD1 (Grad et al., 2014), and TDP-43 (Iguchi et al., 2016). EV fractions isolated from HD brain tissue and injected into wild-type mouse brains induce mHTT pathology and HD-like behavioral phenotypes (Jeon et al., 2016), suggesting that exosome-mediated transmission could be an important pathogenic mechanism that supports long-range propagation.

### Tunneling nanotubes

Tunneling nanotubes (TNTs) are thin, membrane-enclosed extensions that form cytoplasmic bridges between cells across short or long (up to 200 μm) distances (Onfelt et al., 2005). TNTs can be most readily observed in cell monolayers and are defined based on their transient nature, presence of F-actin, and formation above the substratum. TNTs transport diverse intracellular signaling molecules and cargos between cells, including Ca^2+^, RNAs, lipids, proteins, viruses, endosomes and lysosomes, and even large organelles such as endoplasmic reticulum, Golgi, and mitochondria. TNTs generate a direct line of communication between cells under physiological conditions and can be induced by a variety of cell stressors or pathological states (Victoria and Zurzolo, 2017). TNTs allow cells to exchange material that can promote survival, especially in suboptimal or stressful environments. For example, TNTs formed by cells under stressful conditions permit healthier cells to donate functional organelles such as mitochondria, thereby increasing survival in a population of cells (Cho et al., 2012; Islam et al., 2012; Ahmad et al., 2014; Rostami et al., 2017). Similarly, TNTs can transfer damaged proteins and organelles from unhealthy to healthy cells, or from more vulnerable to more resistant cells, in an effort to expedite debris clearance (Rostami et al., 2017; Scheiblich et al., 2021).

The first hint that TNTs could play a role in propagating neurodegenerative disease pathology came from the Zurzolo lab in 2009 describing TNT-mediated transfer of infectious prions between catecholaminergic neuronal cells in cultured monolayers (Gousset et al., 2009). PrP*^Sc^* aggregates were detected within TNTs connecting infected and uninfected neuronal cells, bone marrow-derived dendritic cells, and primary neurons and astrocytes. Interestingly, TNT-mediated transfer of PrP*^Sc^* between primary astrocytes occurred more efficiently than *via* secretion, and PrP*^Sc^* aggregates detected within TNTs were associated with markers of endocytic vesicles (Zhu et al., 2015). Neuronal cells treated with fibrils formed by the N-terminal 480 amino acid residues of mHTT (mHTT_*N*480_), Aβ, tau, or α-synuclein induced TNT formation, and FP-tagged mHTT_*N*480_ proteins were detected inside some of the TNTs (Costanzo et al., 2013; Tardivel et al., 2016; Rostami et al., 2017; Chastagner et al., 2020). Interestingly, transfer of mHTT proteins between cultured neuronal cells was found to occur simultaneously with the MSN-enriched and SUMO E3-like protein Rhes in a SUMO-dependent manner (Sharma and Subramaniam, 2019), suggesting that TNT-like structures facilitate mHTT transfer between the most vulnerable neurons in HD (Figure 3F). Though the hypothesis that pathogenic mHTT aggregates could be disseminated between cells or brain regions *via* TNTs is an attractive one, identification and characterization of TNT-like structures in the brain and the molecular events that lead to TNT formation and cargo transport will help to determine the clinical relevance of these structures in HD and other neurodegenerative diseases.

### Roles for non-neuronal cells

While much HD research to date has focused on the impacts of mHTT aggregation on neuron structure and function, emerging evidence suggests that non-neuronal cells, particularly astrocytes and microglia, are key players in HD neuropathogenesis (Wilton and Stevens, 2020). Reactive gliosis is one of the earliest and most prominent findings in HD patient brains and is recapitulated in many mouse models of neurological disease (Sapp et al., 2001; Faideau et al., 2010; Franciosi et al., 2012; Kraft et al., 2012), suggesting that glial cells play roles at every stage of disease. Reactive astrocytes and microglia alter their morphology, proliferate, and activate gene expression programs that promote inter-cellular communication, removal of toxic debris, and neuronal survival (Burda and Sofroniew, 2014; Hammond et al., 2019). Failure to extinguish these responses once homeostasis is achieved and dysregulation of immune signaling pathways leads to chronic neuroinflammation and development of neurotoxic phenotypes in astrocytes and microglia (Chung et al., 2015; Liddelow et al., 2020).

Glial dysfunction in HD arises directly from mHTT expression or indirectly due to interactions with damaged neurons and other extracellular debris, possibly including mHTT aggregates themselves. Though mHTT inclusions are much more prominent in neurons, mHTT expression has been detected in glia in HD brains (Myers et al., 1991; Sapp et al., 2001; Shin et al., 2005; Benraiss et al., 2021). Reduced appearance of aggregated mHTT in glia may result from increased proteostatic capacity and/or enhanced clearance of intracellular mHTT aggregates *via* ubiquitin-dependent or autophagic pathways in these cell types (Zhao et al., 2016, 2017; Jansen et al., 2017). Targeted expression of mHTT in astrocytes and microglia is sufficient to decrease lifespan and cause HD-like behavioral phenotypes in mice (Bradford et al., 2009, 2010), whereas silencing mHTT expression in astrocytes slows disease onset and rescues neurological phenotypes, striatal atrophy, and synaptic dysfunction *in vivo* (Stanek et al., 2019; Wood et al., 2019). Strategies that lower mHTT expression at the genetic level and improve cellular and behavioral phenotypes are a primary focus of disease-targeted therapeutic development, and have the potential to rescue both neuronal and glial cell defects (Bradford et al., 2009). The ability of astrocytes and microglia to clear mHTT aggregates and dead or dying neurons suggests that these cells may also be viable drug targets (Cho et al., 2019).

A growing body of evidence supports the idea that glia play a central role in spreading mHTT and other pathogenic aggregates in the brain. In *Drosophila*, aggregates formed by mHTT_ex1_ proteins transfer from presynaptic ORNs to postsynaptic PNs in the fly olfactory system only after passage through the cytoplasm of phagocytic glial cell intermediates (Figure 3J) (Donnelly et al., 2020). This circuitous route for mHTT spreading requires expression of Draper (Pearce et al., 2015; Donnelly et al., 2020), a scavenger receptor that regulates key phagocytic pathways in fly glia and other cell types (Figure 3G) (MacDonald et al., 2006; Etchegaray et al., 2016; Ray et al., 2017). The mammalian homolog of Draper, MEGF10, is highly expressed in astrocytes and mediates phagocytic clearance of synapses in healthy and diseased adult mouse brains (Chung et al., 2013; Iram et al., 2016; Shi et al., 2021). Interestingly, MEGF10 binds to complement cascade factor C1q (Iram et al., 2016) and mediates endocytic uptake of Aβ (Singh et al., 2010; Fujita et al., 2020), suggesting a conserved role for this phagocytic receptor in engulfing aggregates. Roles for glia in aggregate transmission have also been described for mutant tau (Asai et al., 2015; Chastagner et al., 2020) and α-synuclein proteins (Lee et al., 2010; George et al., 2019; Dutta et al., 2021; Scheiblich et al., 2021) and could involve dissemination of prion-like aggregates due to inefficient clearance of engulfed aggregates by the phagolysosomal system (Brelstaff et al., 2021).

Accumulation of mHTT in the CSF correlates with mHTT load in the brain and clinical symptoms in HD patients and is currently used in clinical trials to measure effects of HTT lowering strategies in the brain (Tabrizi et al., 2019). mHTT release into the CSF *via* active or passive secretion leads to aggregate clearance by the glymphatic system (Caron et al., 2021), but has the potential to lead to mHTT spreading into the periphery. While relatively understudied compared to mHTT spread in the CNS, there is some evidence to support cell-to-cell transmission of mHTT and other aggregates in non-neuronal tissues. mHTT_ex1_ aggregates are detectable in plasma and circulating blood cells, liver, kidney, muscle, and brain tissues of wild-type mice following parabiosis with HD mice expressing mHTT_ex1_ (Rieux et al., 2020). Furthermore, in a *C. elegans* model of HD, HTT_ex1_ proteins spread bidirectionally between pharyngeal muscle cells and neurons in a process accelerated by increased polyQ length and age (Kim et al., 2017). These findings raise the intriguing possibility that spread of mHTT aggregates outside of neuronal tissues may drive systemic HD pathologies often experienced in later stages of HD (Chuang and Demontis, 2021).

## Prion-like disease mechanisms as a therapeutic target

Currently available treatments for HD patients can temporarily improve quality of life by managing motor, cognitive, and psychiatric symptoms, but to date, no therapy can stop or slow HD progression. Therapies currently approved by the FDA for the treatment of HD include vesicular monoamine transporter 2 inhibitors [tetrabenazine (Xenazine^®^) and deutetrabenazine (Austedo^®^)] that reduce chorea, a symptom that affects ∼90% of HD patients, and antipsychotics and other pharmacological agents that help manage cognitive and behavioral manifestations of HD. Unfortunately, these treatments do not modify the course of HD, often produce adverse side effects, and in some cases even exacerbate HD symptoms. The HD therapeutic pipeline continues to expand as a result of basic and pre-clinical research from the last ∼20 years, with numerous potentially disease-modifying approaches under evaluation in clinical trials (Tabrizi et al., 2020).

Therapeutic avenues with the potential to target prion-like mechanisms of HD include HTT-lowering strategies that prevent mHTT aggregate formation and toxicity, approaches that increase clearance of mHTT aggregates, and interventions that target glial inflammatory and/or phagocytic responses in the degenerating brain. Additional HD therapeutic strategies involve cell reprogramming or replacement therapies to rescue the effects of neuronal loss (Connor, 2018). A limited number of studies suggest some improvement in motor and cognitive functions following intrastriatal grafts of fetal neural stem cells in HD patients (Bachoud-Lévi et al., 2000, 2006); however, evidence for host-to-graft spreading of pathological proteins in HD and PD patient brains (Kordower et al., 2008; Li et al., 2008; Cicchetti et al., 2014) raises significant concerns about the utility of cell replacement therapies in prion-like diseases.

HTT-lowering therapeutic strategies are built upon the idea that reducing mHTT expression can prevent all downstream pathogenic events in HD (Leavitt et al., 2020). Approaches that selectively silence mutant HTT alleles without affecting wtHTT expression are especially attractive in avoiding potential adverse effects associated with loss of normal HTT functions (Murthy et al., 2019). There are currently three major HTT-lowering approaches under clinical development: viral delivery of short-interfering RNA (siRNA) molecules, infusion of allele-specific antisense oligonucleotides (ASOs), and gene editing strategies such as DNA-targeting zinc finger nucleases or CRISPR/Cas9 (Tabrizi et al., 2019; Leavitt et al., 2020). Alternative strategies that could selectively target mHTT and not wtHTT include amplification of proteostatic systems to enhance clearance of toxic mHTT proteins from cells, including the UPS, autophagy, and phagocytosis (Harding and Tong, 2018). The potential for TNTs to deliver protective materials (e.g., functional mitochondria) or eliminate damaged or toxic materials (e.g., aggregates) suggest that these structures could be targeted to promote survival of dysfunctional neurons and/or block aggregate spread (Han and Wang, 2021). Despite recent setbacks in clinical trials, perhaps due to limited knowledge about the optimal timing of intervention (Kingwell, 2021), there remains much hope for HTT-lowering therapies as a viable disease-modifying approach for HD.

The early responses of microglia and astrocytes to neuronal cell damage in all neurodegenerative diseases and traumatic brain injuries suggest that glial cells may be promising therapeutic targets to treat many neurological disorders. Remarkably, many genetic factors associated with increased risk of non-familial forms of AD, PD, and/or ALS are predominantly expressed in astrocytes (e.g., the *APOE4* allele) and microglia (e.g., rare variants of *TREM2*), underscoring the idea that glial cell dysfunction plays a critical role in neurodegeneration. Neuroinflammation is a key driver of pathogenesis, and thus targeting pathways that mediate pro-inflammatory signaling is a major focus of drug development. Immunotherapies that inhibit toxic effects and/or stimulate microglial clearance of aggregates are under evaluation for the treatment of AD, PD, and ALS (Kwon et al., 2020), and could directly interfere with prion-like pathogenic mechanisms. Interest in passive immunization as a therapeutic strategy for neurodegeneration has grown following accelerated FDA approval of the anti-Aβ antibody aducanumab for treatment of AD in 2021, although the effectiveness of this treatment in improving disease outcomes remains controversial (Karran and De Strooper, 2022). Preclinical studies suggest that immunotherapies have the potential to block protein aggregation, facilitate clearance by phagocytic microglia and astrocytes, and prevent cell-to-cell spreading by sequestering pathological proteins associated with nearly every neurodegenerative disease, including mHTT (Snyder-Keller et al., 2010; Butler and Messer, 2011). Interestingly, glial cells could also be employed in cell replacement therapies; indeed, transplantation of healthy astrocytes in the brain is currently undergoing clinical testing as a treatment for ALS (Glass et al., 2016). Intrastriatal transplants of glial progenitor cells improve motor coordination and lifespan in HD mice (Benraiss et al., 2016), suggesting that a similar approach may be useful for HD patients. Though many questions remain to be answered, interventions that can rebalance the beneficial vs. neurotoxic effects of glial cells in the degenerating brain have immense potential in the treatment of HD and other neurodegenerative diseases.

## Concluding remarks

Here, we have provided a comprehensive summary of substantial progress that has been made in the last 10–15 years in identifying prion-like characteristics of mHTT proteins. Though not initially thought of as an important component in the development of monogenic disorders such as HD, many studies now provide compelling evidence to support prion-like behavior of mHTT aggregates and potential links to pathological changes observed in HD patients. Key questions that remain include identifying roles for selectively-vulnerable neuronal and non-neuronal cell populations in aggregate spreading, subcellular structures or organelles that could accommodate aggregate entry or escape, active and passive modes of aggregate transmission, and roles for mHTT aggregate polymorphism and genetic risk factors in the transmissibility and toxicity of pathological mHTT proteins. Further elucidating the molecular mechanisms that mediate inter-cellular and inter-regional spreading of mHTT is critical to expanding our understanding of HD neuropathogenesis and identifying novel targets for treatments that can directly modify the course of HD.

## Author contributions

KD, CC, MF, VR, DS, and DT: literature research and draft manuscript preparation. KD and MP: revision and final manuscript preparation. All authors have reviewed and approved the final version of the manuscript.
